# Differential Denaturation of Serum Proteome Reveals a Significant Amount of Hidden Information in Complex Mixtures of Proteins

**DOI:** 10.1371/journal.pone.0057104

**Published:** 2013-03-22

**Authors:** Vincenzo Verdoliva, Cinzia Senatore, Maria Letizia Polci, Stefania Rossi, Martina Cordella, Giuseppe Carlucci, Paolo Marchetti, Giancarlo Antonini-Cappellini, Antonio Facchiano, Daniela D'Arcangelo, Francesco Facchiano

**Affiliations:** 1 Dip. Ematologia, Oncologia e Medicina Molecolare, Istituto Superiore di Sanità, Roma, Italy; 2 Azienda Ospedaliera Sant' Andrea, Università La Sapienza, Ospedale S. Andrea, Roma, Italy; 3 IV Divisione di Oncologia e Oncologia Dermatologica, Istituto Dermopatico dell'Immacolata, Istituto di Ricovero e Cura a Carattere Scientifico, IDI-IRCCS, Roma, Italy; 4 Lab. Dermatologia Applicata, Istituto Dermopatico dell'Immacolata, Istituto di Ricovero e Cura a Carattere Scientifico, IDI-IRCCS, Roma, Italy; University of Bari Medical School, Italy

## Abstract

Recently developed proteomic technologies allow to profile thousands of proteins within a high-throughput approach towards biomarker discovery, although results are not as satisfactory as expected. In the present study we demonstrate that serum proteome denaturation is a key underestimated feature; in fact, a new differential denaturation protocol better discriminates serum proteins according to their electrophoretic mobility as compared to single-denaturation protocols. Sixty nine different denaturation treatments were tested and the 3 most discriminating ones were selected (TRIDENT analysis) and applied to human sera, showing a significant improvement of serum protein discrimination as confirmed by MALDI-TOF/MS and LC-MS/MS identification, depending on the type of denaturation applied. Thereafter sera from mice and patients carrying cutaneous melanoma were analyzed through TRIDENT. Nine and 8 protein bands were found differentially expressed in mice and human melanoma sera, compared to healthy controls (p<0.05); three of them were found, for the first time, significantly modulated: α2macroglobulin (down-regulated in melanoma, p<0.001), Apolipoprotein-E and Apolipoprotein-A1 (both up-regulated in melanoma, p<0.04), both in mice and humans. The modulation was confirmed by immunological methods. Other less abundant proteins (e.g. gelsolin) were found significantly modulated (p<0.05).

Conclusions: i) serum proteome contains a large amount of information, still neglected, related to proteins folding; ii) a careful serum denaturation may significantly improve analytical procedures involving complex protein mixtures; iii) serum differential denaturation protocol highlights interesting proteomic differences between cancer and healthy sera.

## Introduction

Proteins released in the blood-stream reflect the complex network of functions acting within the tissues. In plasma and serum it is possible to find secretory proteins, immunoglobulins, hormones and peptides acting as long-distance signals, cytokines and short-distance signals, products of cell or tissue damage as consequence of apoptotic or necrotic processes including nucleic acids, aberrant secretory products, like the ones released under pathologic conditions, products of non-human origin, like proteins from bacteria, parasites or other micro-organisms, either pathogens or not [1]. Given its circulatory nature, the blood-stream is therefore an important sources of information regarding the function of peripheral district under either normal or pathologic conditions. It is noteworthy that proteins and peptides released in blood-stream may be directly produced by the pathologic tissue or may represent a reaction of the microenvironment surrounding the pathologic tissue itself. Therefore, analysis of serum/plasmatic proteome may be a key step to study the pathogenetic mechanisms or to achieve early diagnosis of many human diseases, including cancer. Unfortunately, despite the relevant effort spent in the past years, sensitive and reproducible biomarkers for early diagnosis or pre-screening analysis are still lacking for many cancer types and biomarkers discovery from blood samples is still considered a big disappointment [2]. Serum proteome collected and characterized under standardized protocols contains a huge amount of molecules. Further, it was suggested that many potentially useful cancer biomarkers are present in traces, below the detection level or still unrecognized [3]. This makes serum biomarkers discovery a challenging field and a hard task, despite the current improvement of analytical techniques. One difficulty comes from the large concentration range existing between the most abundant and the less abundant proteins in serum, encompassing at least 9 orders of magnitude. Moreover, the most represented proteins (e.g. albumin, haptoglobin, microglobulin, transferrin, immunoglubulins and a few others) account for more than 90% of the total serum proteome and impair the analysis of the remaining 10%. To overcome such problem, depletion or equalizing approaches are usually implemented in order to remove the most abundant proteins and to un-reveal the less abundant proteins often present at concentrations lower than 10 ng/ml [4]–[6]. On the other hand, abundant proteins often carry smaller molecules, therefore the “depletion approach” might remove (totally or in part) the carried signals, altering the actual concentration detected in the blood samples [5], [6]. Furthermore, it was recently reported that different protocols to capture low-abundance proteins may have significantly different efficiencies [7]. Therefore, the practice of serum depletion prior to carry on proteomic analysis is still a controversial issue [6]. An additional weakness of the existing protocols involving electrophoretic sera analysis is related to the intrinsic features of serum proteins and other serum components which make difficult to achieve a complete and reproducible serum protein denaturation. Further, individual serum differences may sum to the reproducibility issues of 2D analysis, making it difficult to compare sera derived from large groups of patients [8], [9]. To overcome the limitations of 2D electrophoresis, many authors follow alternative approaches generally based on gel-free systems, although in most cases the complexity of the developed strategies still makes not easy the analysis of large number of sera. On the other hand, mono-dimensional (1D) electrophoresis, while less informative than 2D-electrophoresis, allows the simultaneous separation of more samples. Therefore, one aim of this study was to compare the proteins detected within the serum proteome under different conditions and to identify the optimal denaturation procedures useful for further serum proteome analysis. In the present study a novel procedure was developed, by investigating many different solubilisation/denaturation procedures consisting of different combinations of chemical or physical factors to improve the electrophoretic separation. The type of denaturing agent was selected taking into account the main factors able to improve the solubility and the solvent accessibility of proteins, namely hydrophobicity, ionic strength, temperature and ultrasounds (shock waves). Each denaturation agent was tested alone or in combination with others in different protocols at increasing concentrations or time-length, with the aim to achieve the optimal protein detection. The developed procedure therefore analyzes serum proteins according to their ability to be resolved by different denaturation protocols. To test the efficacy of this approach, 3 differently denaturing protocols were selected to treat a human serum, consisting of a pool of human sera, and a commercial bovine serum, subsequently analyzed by SDS-PAGE and mass spectrometry. The analysis indicates that serum reveals different protein bands according to different denaturation protocols (we refer to this feature as different “denaturability” of the serum). To verify this intriguing finding, the developed differential denaturation protocol (called TRIDENT since consisting of 3 different denaturation treatments) was applied to sera from a murine model of cutaneous melanoma and human sera from patients affected by early non-metastatic cutaneous melanoma and their proteomes were compared to control murine or normal human sera. Nine and 8 proteins were found differentially expressed (p<0.05) in mice and humans melanoma sera, respectively, compared to the corresponding controls and, 3 of them were found similarly modulated both in the mouse and in human cancer subjects. These data indicate that serum denaturation, an essential step for proteome studies and identification, requires further specific investigation to optimize protein discrimination and identification in complex mixtures such as serum/plasma.

## Materials and Methods

### Electrophoresis

Slab gels (2.4–15% continuous acrylamide-bisacrylamide gradient vertical gels, manually poured into 16×18 cm, thickness 1.5 mm, 15 wells) were generated with a gradient maker (Model 385, Bio-Rad, Hercules, CA) and run with the SE 600 Ruby Apparatus (Hoefer, Inc. Holliston, MA), using fresh solutions, according to standard protocols. The final concentration of the gel reagents used were the following: acrylamide-bisacrylamide 3–0.08%, 125 mM Tris-HCl pH 8.8, 0.1% SDS (w/v), 0.1% ammonium persulphate (w/v), 0.07% TEMED (v/v) in the stacking gel and 600 mM Tris-HCl pH 6.8, 0.078% SDS (w/v), 0.045% ammonium persulphate (w/v), 0.047% TEMED (v/v) in the resolving gradient gel. To avoid any exogenous protein contamination, all procedures involving gel pouring, polymerization and handling were performed in a sterile class 2 cabinet. The sample buffer solution used was defined “high stringency sample buffer” (HSSB) containing, as final concentration, 44 mM tris-HCl pH 6.8, 2% SDS (w/v), 10% glycerol (v/v), 5% 2-β-mercaptoethanol (v/v) and 0.0125% bromophenol blue (w/v); it was prepared as 2× stock solution. In these gels, 120 µg of total proteins were loaded per lane. All samples pre-treated with any of the tested protocols were subjected, immediately before the electrophoretic run, to the following denaturation, as follows: a 2× HSSB was mixed to each sample in 1∶1 ratio, then solution was heated for 7 min in a thermoblock pre-heated at 95°C (Thermomixer Compact by Eppendorf, Hamburg, DE), then immediately placed on ice. Electrophoretic running conditions were: 60 min at 100 Volt constant followed by 220 min at 160 Volt constant (at 15°C under thermostatic control). At the end of the run the whole gel, including the stacking portion, was handled. For some comparative experiments, 25 µg of proteins of the same serum samples were loaded both into Precast Gradient Gels (NuPAGE Novex Bis-Tris, gradient gel, 8×8 cm, thickness 1 mm, from Invitrogen, Carlsbad, CA: running conditions were according to the manufacturer's instructions) and into manually poured gradient gels of the same size (run in vertical electrophoretic chambers from Hoefer). The protein bands were detected in the gels by silver staining protocol as previously described by Shevchenko [10], with the following modifications: time of soaking of the gel in 0.1% AgNO_3_ was 30 min; developer solution was prepared in the dark, using sodium carbonate dissolved immediately before starting the staining procedure and fresh solution of DTT; all steps were carried out at room temperature (20°C); development was stopped by adding 1∶1 volume of 10% citric acid. In some experiments, gels were also stained by Coomassie blue G 250 and R-250 (Bio-Rad). Acrylamide, bisacrylamide and 2-β-mercaptoethanol were from ICN Biomedicals (Irvine, CA); AgNO_3_ was from Merck Eurolab (Lutterworth, Leicestershire, UK). All other chemicals and reagents used, analytical grade, were from Sigma-Aldrich (St. Louis, MO). The three denaturation protocols were applied simultaneously to both control and melanoma sera, to ensure that healthy and cancer sera were subjected to the same protocol.

### Densitometric analysis

Bands densitometry was carried out using the Quantity One Software (Bio-Rad); the stained gels were scanned using a ChemiDoc apparatus (Bio-Rad) equipped with a high resolution digital camera and the photos were saved as file pictures (TIF format). Bands expression was evaluated as normalized optical density and only protein bands with a mean density significantly higher or lower than corresponding controls (p<0.05) were considered differentially expressed.

### Serum pre-treatments: TRIDENT Protocol

FBS (Foetal bovine Serum, from Sigma Aldrich) aliquots and a pool of human sera (from 28 healthy individuals) were subjected to 69 different chemical or physical pre-treatments (PT) to achieve different denaturation, before electrophoretic fractionation. The 69 denaturation treatments are described and summarized in Table S1. They consisted of treatments with salts (0.5 to 5 M NaCl and ammonium bicarbonate), high temperature (37°C heating, 100°C boiling or cycles of freeze and thaw for different time length and/or several times), ultrafiltration with different cut-off (3, 10, 30 kDa, Centricon Centrifuge Filters from Millipore, Billerica, MA), sonication for increasing time length, detergents (1 or 2% of Nonidet 40, TritonX100, Tween 20, SDS) and reducing agents (2-βmercaptoethanol, or ((-)-1,4-Dithio-L-threitol, DTT), minimum 95%, Sigma Aldrich). Each pre-treatment was tested alone or in combination with others. Sodium chloride and ammonium bicarbonate were from Carlo Erba (Milano, IT). All other chemicals used were from Sigma Aldrich.

Both human and bovine serum samples were analyzed by gradient SDS-PAGE and the two denaturation pre-treatments able to resolve the highest number of protein bands were selected, namely PT-14 and PT-64. These denaturation pre-treatments were thereafter compared to a “reference denaturation” treatment, the PT-1, similar to the Laemmli protocol, in order to further highlight any differential electrophoretic mobility of proteins due to the different denaturation achieved with the three different protocols. Differentially denatured serum was therefore subjected to gradient SDS-PAGE analysis and the obtained electrophoretic profiles were depicted in 3 different lanes, corresponding to 3 different pre-treatments: reference/PT-1, PT-14 and PT-64 and thereafter, to simplify, such denaturation treatments were called denaturation 1 (DENT1), denaturation 2 (DENT2) and denaturation 3 (DENT3), respectively. Such electrophoretic analysis was therefore called TRIDENT-SDS-PAGE. Gels were stained by Coomassie blue (R-250 and G-250) or silver nitrate protocols. Silver staining procedure was faster and more sensitive than the Coomassie blue, although less suitable for a precise protein band quantification, therefore silver staining protocol was chosen for the analytical process and then immunological methods were used for validation and more accurate quantification. Stained gels were photographed by a digital camera for densitometric quantification.

### Comparison of DENT1, DENT2 and DENT3 protocols

The portion of the gradient gel higher than 98 kDa was then cut and the proteins present in DENT1, DENT2 and DENT3 lanes were identified by LC-MS/MS.

### Identification of differentially expressed proteins

Each protein DENT1, DENT2 and DENT3 band from the whole gradient gel was measured both in control and melanoma sera. Then densitometric values from DENT1 melanoma bands were compared to values corresponding to DENT1 healthy bands. The same was carried out for DENT2 melanoma *vs* DENT2 healthy and DENT3 melanoma *vs* DENT3 healthy protein bands. The bands significantly (p<0.05) up- or down-regulated in melanoma *vs* control sera in at least one of the 3 denaturation treatments were considered of interest, therefore they were cut, digested and subsequently analyzed by MALDI-TOF/MS. The identification of such differentially expressed proteins was further confirmed by LC-MS/MS analysis and validated by immunological methods (see below). Protein normalization was achieved by loading equal amount of total proteins (by Bradford assay, Bio-Rad) and checked by densitometric comparison with immunoglobulin light chain band. When denaturation methods induced some protein aggregation, samples were re-suspended by passing it through a narrow needle (26 gauge) before loading onto the gel.

### Human sera for electrophoretic studies

Human sera were obtained by peripheral vein puncture under full institutional review board approval and patient consent according to standard clinical protocols. For setting up experiments, the pool of healthy human sera was generated by mixing 2 ml of each individual serum from 28 different healthy individuals, consisting of 14 male and 14 female, with age ranging between 25 and 65 years. Mean age was 42.8±13.2 for male individuals and 44.9±15.9 for female individuals. The hemato-clinical parameters (including glycemia, liver/kidney functionality tests, coagulation and lipidemic assays) for each individual were evaluated and only sera showing values within the physiological range were included. For experiments with individual human samples, sera of 10 melanoma patients (5 males and 5 females), with reported diagnosis of cutaneous melanoma at early or non-metastatic stages (I-IIIB stages according to AJCC (NCCN Clinical Practice Guidelines in Oncology, Melanoma, V.2.2010, www.nccn.org) were compared to those from 10 healthy subjects (5 males and 5 females within the same age range) different from those selected to prepare the pool of healthy human sera.

### Cell culture

B16-F10 mouse melanoma cells were provided by ATCC (ATTC Number: CRL-6475). Cells were maintained in Dulbecco's modified Eagle's medium (DMEM, Gibco BRL, Paisley, UK) in the presence of 10% heat-inactivated calf serum (Gibco). B16-F10 cells were cultured in a CO_2_ incubator (5% CO_2_, 37°C) in plastic culture flasks and cell extracts were prepared as described [11]. Cells were detached by trypsin/EDTA harvesting and viable cells were identified by trypan blue exclusion and counted with a hemocytometer, afterward the number of cells to be injected in mice was re-suspended in PBS (2×10^5^ cells/100 µl), as previously described [12].

### Murine model of melanoma

An *in vivo* mouse primary melanoma growth assay was carried out as previously reported with modifications, according to an accepted animal-study protocol [13]. Six adult male C57BL/6 mice (Jackson Laboratories, Bar Harbor, ME) received 2.5×10^5^ B16-F10 cells dissolved in 100 µl of PBS by subcutaneous injection in the dorsal skinfold. As controls, 6 male mice were injected with PBS: the experiment was repeated 4 times, for a total of 24 mice injected with melanoma cells and 24 with PBS. Eight days after melanoma cell injection, 50–100 µl of blood were collected by tail vein puncture from each control and melanoma injected mouse. Then blood samples were kept for 2 h at room temperature to allow coagulation, and the serum was obtained by centrifugation then divided into aliquots and stored at −80°C. Three weeks after melanoma cell injections, tumours were removed surgically, weighted, fixed in formalin 4% for 48 h and embedded in paraffin.

Mice were anaesthetized by an intraperitoneal injection of ketamine-xylazine (ketamine 80–100 mg/kg and 5–10 mg/kg xylazine). Mice were sacrificed by cervical dislocation.

### Ethics Statements

For the experiments conducted with sera from patients and healthy subjects, each patient gave informed written consent, the sera were obtained by peripheral vein puncture and data were thereafter analyzed anonymously. Sera and data were collected according to the protocol approved by IDI-IRCCS Ethics Committee (http://www.idi.it/comitato-etico.aspx) entitled: “Costruzione di banche sieriche, informatizzazione dei dati ed analisi proteomica” (Reg. N. 2005; 154).

Regarding the experiments involving the use of animals, the experimental procedures were performed as described in the protocol deposited according to Decreto Legislativo 116/92 at the review board of Università Cattolica del Sacro Cuore, Roma, approved with the identification number A39B. Therefore all of the protocols used were approved by the Institutional Animal Care and Use Committee at Università Cattolica del Sacro Cuore, Roma. All mice were fed unrestricted with standard normal rodent chow and all efforts were made to minimize suffering.

### Mass spectrometry analysis

#### MALDI-TOF/MS analysis

In order to perform protein identification, bands differentially expressed were excised from gels, reduced with DTT 10 mM ((-)-1,4-Dithio-L-threitol minimum 95%, Sigma Aldrich) for 45 min at 56°C, alkylated with 55 mM iodoacetamide (Sigma Ultra, Sigma Aldrich) at room temperature in the dark and digested with 0.1 mg/ml trypsin sequencing grade from bovine pancreas (Roche Applied Science, Indianapolis, IN) in 25 mM ammonium bicarbonate (Sigma Aldrich) overnight at 37°C. One microliter of the supernatant was loaded on a 96 wells plate (Applied Biosystem, Life Technologies Corporation, Carlsbad, CA) and analyzed by MALDI- time of flight mass spectrometer (TOF/MS) (MALDI-TOF Voyager-DE STR, Applied Biosystems) after crystallization with α-cyano-4-hydroxycinnamic acid as matrix. Instrument calibration was carried out as described [14]. When necessary, tryptic peptides were desalted by μC18 Zip Tip (Millipore). Spectra were analyzed by Data Explorer TM (Data Explorer Version 4.0.0.0 Copyright© 1997–2000, Applied Biosystem) and Moverz software (m/z - Knexus edition Copyright© 1998–2001 Proteometrics, LLC, New York, NY). Proteins were unambiguously identified by searching a comprehensive non-redundant protein database (human and mouse, NCBI) through MASCOT algorithm (Matrix Science, Peptide Mass Fingerprint). Only protein identifications by mass fingerprinting with score >64 (i.e. p<0.05) were considered significant. Data mass accuracy was 50 ppm, fully tryptic cleavage constraints with the possibility to have one miss cleavage were permitted, and expected modifications were static carbamidomethylation on cysteine residues and methionine oxidation.

#### LC-MS/MS analysis

Peptide mixtures were analyzed by nanoflow reversed-phase liquid chromatography tandem mass spectrometry (RP-LC-MS/MS) using an HPLC Ultimate 3000 (DIONEX, Sunnyvale, CA) connected on line with a linear Ion Trap (LTQ, ThermoElectron, San Jose, CA). Peptides were desalted in a trap column (Acclaim PepMap 100 C18, LC Packings, DIONEX) and then separated in a reverse phase column, a 10 cm long fused silica capillary (Silica Tips FS 360-75-8, New Objective, Woburn, MA), slurry-packed in-house with 5 µm, 200 Å pore size C18 resin (Michrom BioResources, CA). Peptides were eluted using a linear gradient from 96% A (H_2_O with 5% acetonitrile and 0.1% formic acid) to 50%B (acetonitrile with 5% H_2_O and 0.1% formic acid) in 44 min, at 300 nl/min flow rate. Analyses were performed in positive ion mode and the HV Potential was set up around 1.7–1.8 kV. Full MS spectra ranging from m/z 400 to 2000 Da were acquired in the LTQ mass spectrometer operating in a data-dependent mode in which each full MS scan was followed by five MS/MS scans where the five most abundant molecular ions were dynamically selected and fragmented by collision-induced dissociation (CID) using a 35% normalized collision energy. Target ions already fragmented were dynamically excluded for 30 s. Tandem mass spectra were matched against SWISSPROT database and through SEQUEST algorithm [15] incorporated in Bioworks software (version 3.3, Thermo Electron) using fully tryptic cleavage constraints with only one miss-cleavage allowed, static carbamidomethylation on cysteine residues and methionine oxidation as variable modification. Data were searched with 1.5 Da and 1 Da tolerance respectively for precursor and fragment ions. A peptide was considered legitimately identified when it achieved cross correlation scores of 1.5 for [M+H]1+, 2.0 for [M+2H]2+, 2.5 for [M+3H]3+, and a peptide probability cut-off for randomized identification of p<0.001.

### Validation by immunological methods

In Western Blot (WB) analyses, human sera from healthy and melanoma individuals were treated with TRIDENT protocol and fractionated electrophoretically on gradient gel: 120 µg of serum proteins per lane were loaded in the gel then blotted onto nitrocellulose membrane (Amersham Biosciences, Uppsala, SE). After blocking for 1 h with 5% milk/PBS (low fatty acid milk powder from Sigma Aldrich solubilised in PBS without calcium and magnesium, PBS^−^, pH 7.2), the membrane was incubated for 75 min with a goat primary antibody (diluted 1∶1000 in 2% milk/PBS) against human α2MG (Sigma Aldrich). The membrane was then washed 3 times for 7 min each with 0.1% Tween 20-PBS (T-PBS), incubated for 1 h with secondary antibody (anti-goat HRP from Santa Cruz Biotechnology Inc., Santa Cruz, CA, diluted 1∶10000 in 2% milk/PBS) and washed again as before. Finally, the immunoreactions were visualized by ECL reagents (Amersham Biosciences). All WB experiments were repeated at least 3 times. Protein loading was checked by Ponceau Red (Bio-Rad) staining of membranes before blocking.

In dot blot analyses, human sera from 10 healthy and 10 melanoma individuals were loaded onto nitrocellulose membrane (50 µg of proteins for each spot, repeated in duplicate). All melanoma patients were selected at early, non-metastatic stage. After blocking for 30 min with 5% milk/PBS, the membrane was incubated for 1 h with rabbit primary antibodies (1∶1000 in 2% milk/PBS)

against human α2MG, human Apo E or Apo A1 (Abcam, Cambridge, UK). Then, the membrane was washed 3 times with 0.1% T-PBS and incubated for 1 h with secondary antibody as for the WB experiments. The signal was visualized with ECL method according to the manufacturer's instructions. All dot blot experiments were repeated at least 3 times. Protein loading was checked by Ponceau Red staining of membranes before blocking.

### Bioinformatic analysis of TRIDENT analyzed versus 2DPAGE analyzed sera

Serum proteins identified by TRIDENT followed by LC-MS/MS analysis were compared to the results obtained by 2D-PAGE analyses of sera reported in the World-Wide 2D Gel-based Proteomics Databases (Expasy World-2DPAGE Portal, a dynamic Portal to query simultaneously World-Wide 2D Gel-based Proteomics Databases: http://world-2dpage.expasy.org/portal) and to the results reported in literature (PubMed).

### Statistical analysis

The results were expressed as mean ± standard error of the mean. Student's two tails t-test was carried out and P values<0.05 were considered significant.

## Results

### Human pool sera electrophoretic analysis

**Figure 1:**
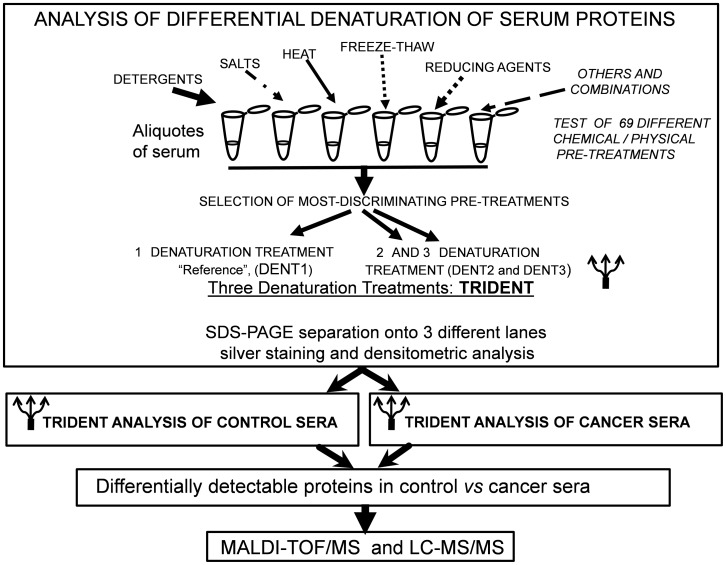
The differential serum denaturation protocol. Schematic representation of chemical-physical pre-treatments of sera and following analytical procedures.

**Figure 2:**
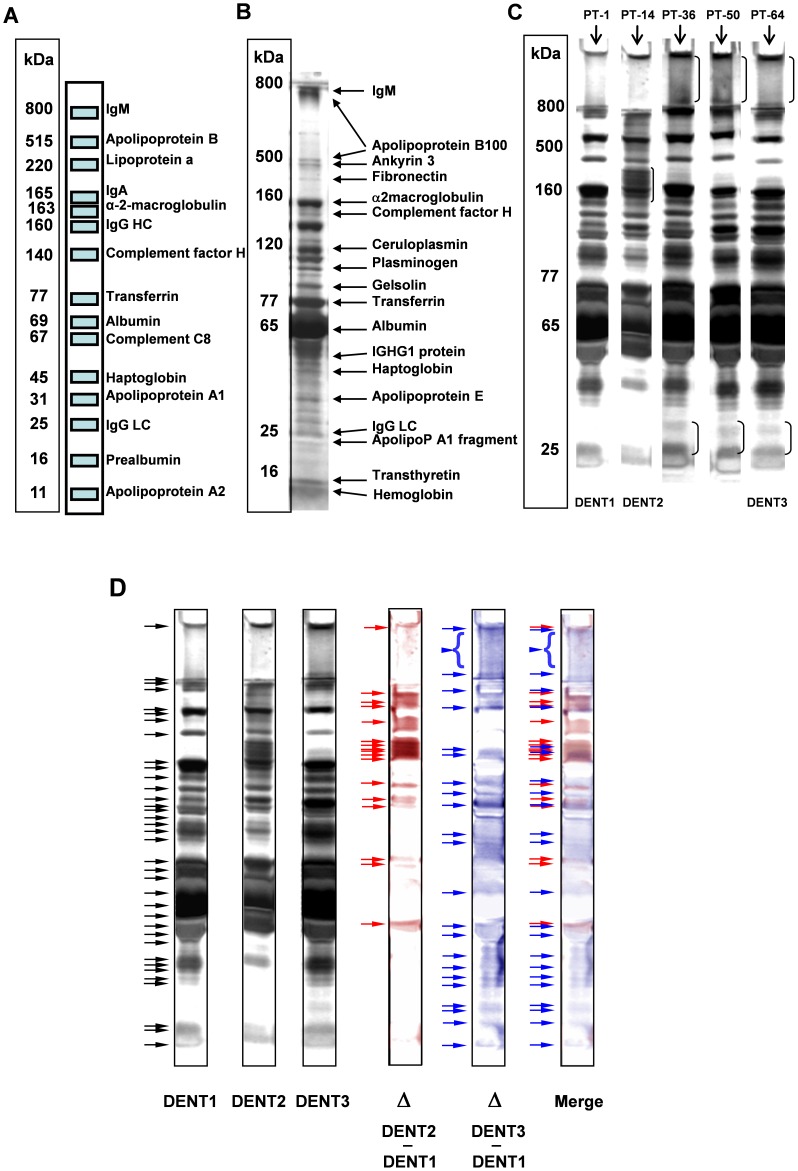
Effects of differential denaturation on serum electrophoretic separation. A: The serum protein pattern as described in literature and as obtained with DENT1 protocol in a 16×18 cm gradient SDS-PAGE (2.4–15%), shown in panel B, by MALDI-TOF/MS analysis of cut and digested protein bands. C: Representative electrophoretic separation of human pooled sera derived from the most discriminating sample pre-treatments (described in Table S1, thereafter defined as DENT2 and DENT3 treatments) compared to the reference treatments (DENT1). Samples were run on a manually poured gradient slab gel (2,4–15%): 120 µg of proteins loaded per lane. Asterisks indicate some of the protein bands undetectable or less detectable in DENT1 but evident in the pre-treatments specifically developed in the present study. D: Graphical representation of the improvement of protein band resolution and discrimination after DENT2 or DENT3 pre-treatments compared to the DENT1. The protein pattern identified as Merge indicates the gain of protein band detection and discrimination.

A preliminary study was first carried out to investigate the SDS-PAGE analysis of serum proteins using a pool of human sera from 28 healthy individuals and a commercial bovine serum (see Methods). Serum was used as whole, i.e. without any depletion step. Sixty-nine different chemical and physical denaturing pre-treatments (see Table S1) were carried out on the pooled sera and SDS-PAGE analysis was performed. The 3 DENaturation Treatments (TRIDENT) showing the highest band discrimination power and sensitivity were then selected. A schematic diagram showing the whole procedure is summarized (Figure 1). The pre-treatment 1 (PT-1) protocol, corresponding to 1∶1 dilution with distilled water followed by denaturation according to standard Laemmli procedure, was considered a “reference” and thereafter identified as “DENT1”. Two additional denaturation treatments showing the highest protein bands discrimination power in SDS-PAGE were the PT-14 and PT-64, as described in Table S1, and were then identified as DENT2 and DENT3 (Figure 2). Data shown in Figure 2 indicate that a markedly different protein pattern is detectable by gradient SDS-PAGE in identical serum samples undergoing different DENTs. In fact, in many cases different denaturation pre-treatments lead to different, reproducible, electrophoretic patterns (see asterisks in Figure 2). In order to compare the pattern of separated proteins with the known serum proteome, the proteins separated by DENT1 were excised and subjected to MALDI-TOF/MS identification. Figures 2A and 2B show the protein bands reproducibly identified by SDS-PAGE under conventional denaturation (DENT1) followed by MALDI-TOF/MS, compared to the serum protein pattern known from the literature. [16]–[20]. When human serum was subjected to additional denaturation pre-treatments, a different electrophoretic pattern was reproducibly observed. In Figure 2C only a few representative pre-treatments selected from the 69 tested are shown, and protein bands differently detected among these lanes, compared to the reference DENT1, are marked by asterisks. Table 1 summarizes the number of total protein bands discriminated through 3 different DENTs selected as above mentioned, on the same serum and demonstrates that TRIDENT protocol may significantly improve the SDS-PAGE separation of whole undepleted serum proteins. In fact, by using a silver staining protocol to visualize the proteins, 17 additional bands are detectable with DENT2 and 23 additional bands are detectable with DENT3, when compared to DENT1. Therefore, while the single denaturation treatment (DENT1) was able to discriminate 36 different serum protein bands (black arrows in Figure 2D), the TRIDENT protocol detected an additional set of at least 40 protein bands in the same serum sample (blue and red arrows in Figure 2D). The protein sensitivity of the detection method was estimated to be lower than 5 ng/band. Table 2 reports a list of some proteins detected by TRIDENT protocol and identified by MALDI-TOF/MS. It is noteworthy that TRIDENT-SDS-PAGE was able to reveal some proteins in human serum, namely H2 MHC-I antigen, the IGHG1 protein and Q10 α-chain, which are undetectable or extremely difficult to be identified in serum fractionated with conventional procedure followed by MALDI-TOF/MS analysis [21], [22].

**Table 1:** Human serum protein bands resolution.

Pre-treatment	DENT1	DENT2	DENT3	DENT1+DENT2+DENT3
**Total protein bands detectable**	36±1	53±1	59±2	36+53+59 = 148
**Newly detected bands (** ***vs*** ** DENT1)**		17	23	
**Number of different bands detectable**	36±1	36+17 = 53	36+23 = 59	36+17+23 = 76

Human pool serum bands resolution in the gradient vertical slab gel 2.4–15%, 16×18 cm, and effects of TRIDENT analysis on the gel bands discrimination. It is noteworthy that the total numbers of bands detectable under DENT2 (53±1) or DENT3 (59±2) pre-treatment reflect the improvement of bands resolution compared to the standard denaturation protocol DENT1 (36±1) (see also Figure 2D).

**Table 2:** Some serum proteins identified by TRIDENT-SDS PAGE compared to bibliographic references.

						MASS ERRORS											
PROT.	S	%C	#P	ANN.	DENT	sequence	Observed	Mr(expt)	Mr(calc)	ppm	Miss	M SC	#MVS	#MVM	MW T	MW O	CONC.
*Alpha-2 macroglobulin*	H&M	29	27	Accession: P0123 (H): Q61838 (M)	2 & 3	K.AESPVFVQTDKPIYKPGQIVK.F	2344.31	2343.306	2343.28	10	0	191	56	27	165	∼170 KDa	Plasma/serum: 1–6 g/L [36], [37]
						K.KIEHSFEVK.E	1116.6	1115.593	1115.6	−4	1						
						K.EYVLPKFEVIIK.M	1477.85	1476.84	1476.86	−13	1						
						K.TMAFLEEELPITACGVYTYGKPVPGLVTLR.V	3325.84	3324.829	3324.71	35	0						
						K.EEGTGIELTGIGSCEIANALSKLK.F	2490.28	2489.275	2489.27	3	1						
						K.VNTNYRPGLPFSGQVLLVDEK.G	2346.28	2345.275	2345.24	16	0						
						R.IHYLLNEDIMK.N	1388.71	1387.704	1387.72	−9	0						
						R.IHYLLNEDIMKNEK.T	1759.92	1758.911	1758.9	8	1						
						K.AAPLSLCALTAVDQSVLLLKPEAK.L	2508.41	2507.403	2507.4	0	0						
						K.LSPQSIYNLLPGK.T	1429.8	1428.789	1428.8	−6	0						
						K.HSLGDNDAHSIFQSVGINIFTNSK.I	2601.3	2600.296	2600.26	13	0						
						R.FCQEFQHYPAMGGVAPQALAVAASGPGSSFR.A	3238.63	3237.62	3237.51	33	0						
						R.AMGVPMMGLDYSDEINQVVEVR.E	2453.18	2452.176	2452.14	13	0						
						R.AMGVPMMGLDYSDEINQVVEVR.E	2469.19	2468.185	2468.14	19	0						
						K.VPDTITEWK.A	1088.56	1087.548	1087.56	−6	0						
						K.ATVLNYMSHCIQIR.V	1705.86	1704.851	1704.84	4	0						
						R.VDLEISPDFLAVPVGGHENSHCICGNER.K	3121.59	3120.586	3120.44	47	0						
						K.AINYLISGYQR.Q	1297.69	1296.682	1296.68	0	0						
						K.AFAQAQSHIFIEK.T	1489.79	1488.78	1488.77	5	0						
						K.THITNAFNWLSMK.Q	1562.76	1561.75	1561.77	−13	0						
						K.GGVDDEVTLSAYITIALLEMPLPVTHSAVR.N	3167.68	3166.671	3166.66	4	0						
						R.NALFCLETAWASISQSQESHVYTK.A	2770.38	2769.369	2769.31	22	0						
						K.RSELLESLNK.D	1188.66	1187.657	1187.65	5	1						
						K.DAVKEEDSLHWQRPGDVQK.V	2237.11	2236.102	2236.09	6	1						
						K.EEDSLHWQRPGDVQK.V	1823.87	1822.861	1822.86	0	0						
						K.ALSFYQPR.A	981.512	980.5046	980.508	−3	0						
						K.MVSGFIPMKPSVK.R	1436.8	1435.796	1435.76	27	0						
*Gelsolin*	H&M	25	22	Accession: Q5T0I2 (H): P13020 (M)	3	K.AGKEPGLQIWR.V	1254.7	1253.691	1253.69	2	1	117	92	22	86	∼150 KDa	Cytoskeletal protein [38], also in plasma-serum 0.1–0.3 mg/ml [41]
						K.EPGLQIWR.V	998.547	997.5399	997.535	5	0						
						R.EVQGFESSTFSGYFK.S	1712.77	1711.766	1711.77	−4	0						
						K.HVVPNEVVVQR.L	1275.72	1274.71	1274.71	0	0						
						K.GIRDNER.S	859.434	858.4264	858.431	−5	1						
						R.SEDCFILDHGR.D	1348.6	1347.594	1347.59	5	0						
						R.SEDCFILDHGRDGK.I	1648.74	1647.734	1647.73	2	1						
						K.QANMEER.K	877.389	876.3819	876.376	7	0						
						R.QTQVSVLPEGGETPLFK.Q	1829.96	1828.951	1828.96	−4	0						
						R.VPFDAGTLHTSTAMAAQHGMDDDGTGQK.Q	2891.34	2890.333	2890.25	29	0						
						R.IEGSNKVPVDPATYGQFYGGDSYIILYNYR.H	3399.65	3398.644	3398.65	−1	1						
						K.VPVDPATYGQFYGGDSYIILYNYR.H	2771.33	2770.322	2770.33	−2	0						
						R.DGGQTAPASIR.L	1072.53	1071.525	1071.53	−5	0						
						K.KMDAHPPR.L	951.488	950.4807	950.476	5	1						
						K.MDAHPPR.L	823.386	822.3785	822.381	−3	0						
						K.MDAHPPR.L	839.397	838.3899	838.376	17	0						
						R.LFACSNR.I	867.414	866.4069	866.407	0	0						
						R.LFACSNRIGR.F	1193.6	1192.596	1192.61	−14	1						
						K.RYIETDPANR.D	1234.64	1233.628	1233.61	14	1						
						R.YIETDPANR.D	1078.52	1077.515	1077.51	5	0						
						R.YIETDPANRDR.R	1349.63	1348.621	1348.64	−12	1						
						R.TPITVVR.Q	785.484	784.4767	784.481	−5	0						
*IGHG1 protein*	H	28	8	Accession: Q6PJA4	3	-.STKGPSVFPLAPSSK.S	1502.78	1501.774	1501.81	−27	1	69	61	8	52	∼100 KDa	Plasma-serum protein [39]
						K.GPSVFPLAPSSK.S	1186.64	1185.637	1185.64	−2	0						
						K.DTLMISR.T	835.441	834.4332	834.427	8	0						
						R.TPEVTCVVVDVSHEDPEVK.F	2139.05	2138.045	2138.02	12	0						
						K.FNWYVDGVEVHNAK.T	1677.81	1676.802	1676.79	4	0						
						R.EPQVYTLPPSR.E	1286.68	1285.674	1285.67	6	0						
						R.EPQVYTLPPSREEMTK.N	1904.97	1903.959	1903.93	13	1						
						R.WQQGNVFSCSVMHEALHNHYTQK.S	2801.32	2800.316	2800.26	20	0						
*C4B5*	H	44	13	Accession: Q6U2L9	3	R.LPMMRSCEQR.A	1307.66	1306.651	1306.59	43	1	95	77	13	48	∼150 KDa	Plasma and serum : range 0.2–0.5 g/L [40]
						R.EPFLSCCQFAESLR.K	1743.78	1742.77	1742.78	−3	0						
						R.DKGQAGLQR.A	972.506	971.4989	971.515	−16	1						
						R.ALEILQEEDLIDEDDIPVR.S	2225.14	2224.13	2224.11	9	0						
						K.GLCVATPVQLR.V	1213.65	1212.644	1212.66	−17	0						
						R.EFHLHLR.L	951.5	950.4931	950.509	−16	0						
						R.GSFEFPVGDAVSK.V	1339.64	1338.633	1338.65	−9	0						
						K.EGAIHREELVYELNPLDHR.G	2290.15	2289.147	2289.15	−2	1						
						R.TLEIPGNSDPNMIPDGDFNSYVR.V	2551.2	2550.197	2550.17	11	0						
						R.VTASDPLDTLGSEGALSPGGVASLLR.L	2483.34	2482.334	2482.29	17	0						
						R.YLDKTEQWSTLPPETK.D	1935.94	1934.933	1934.96	−15	1						
						R.KADGSYAAWLSR.D	1324.65	1323.646	1323.66	−8	1						
						K.ADGSYAAWLSRDSSTWLTAFVLK.V	2545.17	2544.164	2544.26	−40	1						
*H2-MHC-I*	H&M	29	9	Accession: P01898	2 &3	R.YFETSVSRPGLGEPR.F	1694.85	1693.843	1693.84	1	0	72	49	9	37	>800 KDa	Membrane,also secreted [42]
						R.APWMEQEGPEYWER.E	1807.77	1806.765	1806.77	−1	0						
						R.AKGNEQSFHVSLR.T	1472.74	1471.733	1471.75	−13	1						
						K.GNEQSFHVSLR.T	1273.63	1272.623	1272.62	2	0						
						R.DYIALNEDLK.T	1193.62	1192.61	1192.6	10	0						
						R.KWEQAGAAEYYR.A	1471.7	1470.689	1470.69	0	1						
						R.YLELGKETLLR.T	1334.75	1333.742	1333.76	−14	1						
						R.TDPPKTHVTHHPGSEGDVTLR.C	2281.1	2280.095	2280.12	−13	1						
						K.THVTHHPGSEGDVTLR.C	1742.85	1741.842	1741.85	−4	0						
Apolipoprotein A1 protein	H	72	25	Accession: CAA00975	3	-.DEPPQSPWDR.V	1226.55	1225.538	1225.54	1	0	306	47	25	38	∼50 KDa	Plasma and serum 1.0–2.3 g/L [41]
						-.DEPPQSPWDRVK.D	1453.72	1452.712	1452.7	9	1						
						K.DLATVYVDVLKDSGR.D	1650.89	1649.878	1649.86	9	1						
						K.DSGRDYVSQFEGSALGK.Q	1815.88	1814.871	1814.84	15	1						
						R.DYVSQFEGSALGK.Q	1400.66	1399.65	1399.66	−8	0						
						K.LLDNWDSVTSTFSK.L	1612.78	1611.774	1611.78	−2	0						
						K.VQPYLDDFQK.K	1252.62	1251.614	1251.61	0	0						
						K.VQPYLDDFQKK.W	1380.71	1379.702	1379.71	−4	1						
						K.KWQEEMELYR.Q	1411.68	1410.675	1410.66	11	1						
						K.WQEEMELYR.Q	1283.59	1282.579	1282.57	11	0						
						K.WQEEMELYR.Q	1299.59	1298.586	1298.56	20	0						
						R.QKVEPLR.A	869.513	868.5061	868.513	−8	1						
						K.VEPLRAELQEGAR.Q	1467.79	1466.786	1466.78	1	1						
						R.AELQEGAR.Q	873.443	872.4356	872.435	0	0						
						R.QKLHELQEK.L	1152.62	1151.613	1151.63	−15	1						
						K.LHELQEK.L	896.465	895.4579	895.476	−21	0						
						K.LSPLGEEMR.D	1031.52	1030.514	1030.51	2	0						
						K.LSPLGEEMRDR.A	1318.62	1317.618	1317.63	−13	1						
						R.AHVDALR.T	781.432	780.4246	780.424	1	0						
						R.THLAPYSDELR.Q	1301.66	1300.653	1300.64	9	0						
						R.LEALKENGGAR.L	1157.63	1156.622	1156.62	1	1						
						R.LAEYHAK.A	831.419	830.4121	830.429	−20	0						
						K.ATEHLSTLSEK.A	1215.62	1214.614	1214.61	0	0						
						K.AKPALEDLR.Q	1012.58	1011.575	1011.57	4	0						
						K.VSFLSALEEYTK.K	1386.71	1385.699	1385.71	−6	0						
Apolipoprotein E	H&M	46	20	Accession: P02649 (H); Q4FK40 (M)	2 &3	R.FWDYLR.W	899.451	898.4437	898.434	11	0	203	43	20	36	>800 KDa	Plasma and serum: 30–40 mg/L [43], [44]
						K.ELEEQLGPVAEETR.A	1599.84	1598.83	1598.78	32	0						
						K.ELEEQLGPVAEETRAR.L	1826.95	1825.939	1825.92	12	1						
						1 R.LGKEVQAAQAR.L	1170.67	1169.667	1169.65	13	1						
						R.LGADMEDLR.N	1019.48	1018.472	1018.48	−3	0						
						R.LGADMEDLRNR.L	1289.65	1288.642	1288.62	17	1						
						R.NEVHTMLGQSTEEIR.A	1743.88	1742.876	1742.83	29	0						
						R.LSTHLRK.M	854.528	853.5203	853.513	8	1						
						R.ERLGPLVEQGR.Q	1253.72	1252.717	1252.69	22	1						
						R.LGPLVEQGR.Q	968.57	967.5629	967.545	18	0						
						R.TANLGAGAAQPLR.D	1239.71	1238.705	1238.67	26	0						
						R.TANLGAGAAQPLRDR.A	1510.83	1509.824	1509.8	15	1						
						R.AQAFGDR.I	764.391	763.3834	763.361	29	0						
						R.AQAFGDRIR.G	1033.57	1032.562	1032.55	15	1						
						R.GRLEEVGNQAR.D	1228.66	1227.648	1227.63	13	1						
						R.DRLEEVR.E	916.483	915.476	915.477	−1	1						
						R.EHMEEVR.S	929.435	928.4279	928.407	22	0						
						R.SKMEEQTQQIR.L	1377.71	1376.704	1376.67	24	1						
						K.MEEQTQQIR.L	1162.59	1161.578	1161.54	28	0						
						R.LQAEIFQAR.L	1075.61	1074.601	1074.58	18	0						
*HP protein (Haptoglobin)*	H	48	13	Accession: Q6NSB4	3	R.ILGGHLDAK.G	923.521	922.5139	922.524	−10	0	136	45	13	32	∼40 KDa	Plasma and serum: 0.3–2.5 g/L [45], [46]
						K.GSFPWQAK.M	920.457	919.4494	919.455	−6	0						
						K.DIAPTLTLYVGKK.Q	1418.81	1417.804	1417.82	−10	1						
						K.QKVSVNER.V	959.528	958.5202	958.52	1	1						
						R.VMPICLPSKDYAEVGR.V	1834.93	1833.924	1833.91	7	1						
						K.DYAEVGR.V	809.37	808.3631	808.372	−10	0						
						R.VGYVSGWGR.N	980.524	979.5168	979.488	30	0						
						K.FTDHLK.Y	760.389	759.3812	759.392	−14	0						
						K.YVMLPVADQDQCIR.H	1707.83	1706.825	1706.81	7	0						
						R.HYEGSTVPEKK.T	1274.63	1273.626	1273.63	−3	1						
						K.SPVGVQPILNEHTFCAGMSK.Y	2172.06	2171.05	2171.05	0	0						
						K.SCAVAEYGVYVK.V	1345.64	1344.63	1344.64	−6	0						
						K.VTSIQDWVQK.T	1203.62	1202.617	1202.63	−10	0						
*Transthyretin*	H&M	72	7	Accession: P02766(H), Q5M9K1(M)	3	-.KCPLMVK.V	875.506	874.499	874.477	25	1	90	44	7	16	∼30 KDa	Serum, 0.2 g/L [47]
						R.GSPAINVAVHVFR.K	1366.76	1365.752	1365.75	0	0						
						R.GSPAINVAVHVFRK.A	1494.83	1493.825	1493.85	−14	1						
						R.KAADDTWEPFASGK.T	1522.72	1521.708	1521.71	−1	1						
						K.AADDTWEPFASGK.T	1394.61	1393.607	1393.62	−6	0						
						K.TSESGELHGLTTEEEFVEGIYKVEIDTK.S	3140.55	3139.538	3139.51	10	1						
						K.ALGISPFHEHAEVVFTANDSGPR.R	2451.18	2450.177	2450.2	−9	0						

Some of human and murine serum proteins identified with MALDI-TOF/MS are listed. For each identified protein, the following information is reported: PROT. (protein name), S (source, H = human, M = mouse), %C (percentage coverage), #P (number of unique peptides identified), ANN (annotations with theoretical MW and NCBI protein accession number), DENT (denaturation treatment used), mass errors for each sequence analysed, MSC (mass score), #MVS (number of mass values searched), #MVM (number of mass values matched), MW T and MW O (theoretical and observed molecular weights, respectively) and CONC (the serum/plasma concentration levels known by literature).

### Optimization of TRIDENT protocol and LC-MS/MS analysis

**Figure 3:**
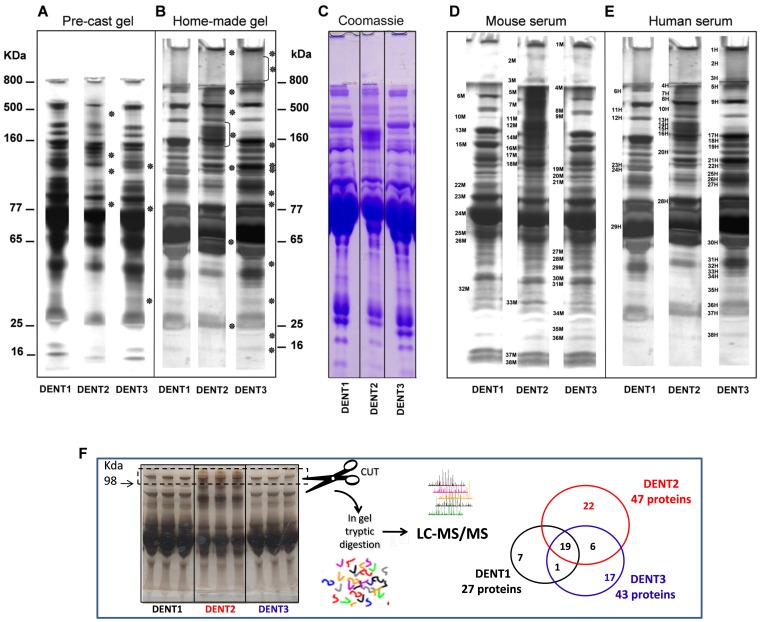
Increased protein band discrimination in serum subjected to differential denaturation. A and B: TRIDENT-SDS-PAGE using pre-cast and manually poured gels, respectively. Representative electrophoretic human pooled sera patterns are shown, after application of TRIDENT differential denaturation protocol, run on pre-cast gradient gel (4–12%) and manually poured 2.4–15% gradient gel (in both cases 8×8 cm gels, 1 mm thickness). Equal amount of serum proteins (25 µg) were loaded onto both types of gel and asterisks indicate some of protein bands newly detectable in consequence of the pre-treatment compared with DENT1 pre-treatment (reference). C: TRIDENT analysis of human serum as reported in C was stained by Coomassie Blue G-250; D and E: representative TRIDENT electrophoretic patterns of healthy murine and healthy human sera, respectively. The bands of interest are identified with a number and a letter (M or H), and the identification of those differentially expressed is reported in Tables 4, 5 and 6. F: Human serum was analyzed by TRIDENT analysis (run in triplicate). Fragment of >98 kDa was cut, trypsin-digested and proteins identified through LC-MS/MS analysis (LTQ).

In order to select the most effective polyacrylamide gradient according to the resolution power of our electrophoretic protocols, we fractionated human pooled sera proteins, pre-treated according to the TRIDENT protocol, on 8×8 cm manually generated gradient gels (2.4–12%, 2.4–15%, 4–12%, 4–15%). In these experiments, the most sensitive electrophoretic separation was achieved by using 2.4–15% acrylamide-bisacrylamide continuous gradient gel. A representative SDS-PAGE of human serum is reported (Figures 3A and 3B). The protein band resolution, evaluated as the number of protein bands detectable on the gel, is summarized in Table 3, showing that DENT1, DENT2 and DENT3 protocols achieve quantitatively (Table 3) and qualitatively (Figures 2 and 3) different results. The differences between DENT1, DENT2 and DENT3 were detectable by silver and Coomassie blue G-250 staining (Figure 3). To confirm the improved protein pattern resolved by the TRIDENT protocol, sera from healthy mice and from healthy humans were analyzed and bands were cut and subjected to MALDI-TOF/MS and LC-MS/MS for protein identification (Figures 3D and 3E, respectively, and Tables 4, 5 and 6). The higher protein discrimination of DENT2 and DENT3 compared to DENT1 was further confirmed by LC-MS/MS analysis of the bands migrated at >98 kDa molecular weight. In this small portion of gel, LC-MS/MS identified 27 different proteins in DENT1 treated lanes, 47 different proteins in DENT2 treated lanes and 43 different proteins in DENT3 treated lanes and results from our analyses were compared to protein bands database reported at the Expasy World-2DPAGE Portal and to the results from proteomics studies present in literature (PubMed) (see Figure 3F and Table S2). Such analysis indicated that 23 proteins identified by TRIDENT analysis followed by mass spectrometry were absent in the serum proteome database and never reported as serum/plasma components in studies published on PubMed, confirming a significant sensitivity increase with TRIDENT as compared to 2D-PAGE. This confirmed also that DENT2 and DENT3 treatments were able to improve the detection of serum proteins *vs* DENT1 and further demonstrated that different denaturation protocols applied to the same serum samples may reveal more and different protein data sets, depending on the type of denaturation selected.

**Table 3:** Human serum protein bands resolution differentially denatured.

	DENT1	DENT2	DENT3
**Total protein bands detectable**	35±1	42±1	46±1

Human serum protein bands resolution differentially denatured.

Estimation of serum protein bands resolution of serum differentially denatured with 3 different protocols, run onto 8×8 cm 2.5–15% gradient gels, visualized by silver staining protocol. Data are reported as mean ±SD.

**Table 4:** MALDI-TOF/MS analysis of differentially expressed bands in melanoma patients.

Human Band	p value	State change	Prot ID	AC #
**4H**	0.0047	Up-regulated in Mel	Apo B	P04114
**16H**	0.0255	Up-regulated in Ctrl	Albumin	Q56G89
**18H**	0.0286	Up-regulated in Ctrl	α2-MacroGlobulin	P01023
**19H**	0.04306	Up-regulated in Ctrl	Ceruloplasmin	Q1L857
**20H**	0.0320	Up-regulated in Ctrl	α2-MacroGlobulin	P01023
**27H**	0.0402	Up-regulated in Ctrl	α-Fetoprotein	P02771
**28H**	0.0520	Up-regulated in Ctrl	α-Fetoprotein	P02771
**31H**	0.0164	Up-regulated in Mel	Apo E	P02649
**34H**	0.0096	Up-regulated in Mel	Apo A1	P02647
**36H**	0.0419	Up-regulated in Mel	Transthyretin	P02766

MALDI-TOF/MS analysis of differentially expressed bands in melanoma patients.

Differentially expressed bands by TRIDENT-SDS-PAGE identified by MALDI-TOF/MS in human sera from cancer patients compared to the healthy controls. P value means the significance between densitometry of control (Ctrl) bands *vs* melanoma bands (Mel), whose modulation is reported as State change. Prot ID describes the name of the protein and AC# the accession number.

**Table 5:** LC-MS/MS analysis of differentially expressed bands in melanoma patients.

Reference	P(pro)	Score	Coverage	MW	Accession	Peptide
transthyretin [H. sapiens]	5.00E-14	50.29	48.30	15877.1	4507725	5
alpha-2-macroglobulin precursor [H. sapiens]	2.22E-15	440.35	36.60	163188.3	66932947	44
apolipoprotein A 1 preproprotein [H. sapiens]	6.99E-14	230.29	52.80	30758.9	4557321	23
apolipoprotein E precursor [H. sapiens]	3.54E-07	60.21	16.40	36131.8	4557325	6
Serum albumin [H. sapiens]	1.11E-14	538.29	75.2	69321.6	4502027	54
apolipoprotein B precursor [H. sapiens]	5.33E-14	740.33	23.30	515209.6	105990532	74
ceruloplasmin precursor [H. sapiens]	5.37E-10	100.30	14.00	122127.6	4557485	10

Some of human serum proteins whose expression was significantly different in melanoma *vs* control sera were further identified by LC-MS/MS. For each identified protein, the following information are reported: Reference = name as reported in annotations; P(pro) = peptide probability; Score; Coverage = percentage of coverage; MW = theoretical molecular weight; Accession = NCBI protein accession number; Peptide = number of unique peptides identified.

**Table 6:** MALDI-TOF/MS analysis of differentially expressed bands in melanoma carrying mice.

Mouse Band	p value	State change	Prot ID	AC #
**15M**	0.0074	Up-regulated in Ctrl	α2-Macroglobulin	Q61838
**21M**	0.0476	Up-regulated in Mel	Complement Factor B	Q3UEG8
**22M**	0.0036	Up-regulated in Mel	Gelsolin	P13020
**23M**	0.0167	Up-regulated in Ctrl	Transferrin	Q921I1
**27M**	0.0093	Up-regulated in Mel	Albumin	P07724
**28M**	0.0194	Up-regulated in Mel	Apo A-VI	Q91XF
**29M**	0.03568	Up-regulated in Mel	MHC I Antigen H2Q10	P01898
**32M**	0.0056	Up-regulated in Mel	Apo E	Q4FK40
**35M**	0.0295	Up-regulated in Mel	Apo A1	Q00623

Differentially expressed bands by TRIDENT-SDS-PAGE identified by MALDI-TOF/MS in murine sera from cancer animal compared to the healthy controls. P value means the significance between densitometry of control (Ctrl) bands *vs* melanoma bands (Mel), whose modulation is reported as State change. Prot ID describes the name of the protein and AC# the accession number.

### TRIDENT protocol to highlight serum proteome differences between healthy and cancer sera

#### Human melanoma model study

**Figure 4:**
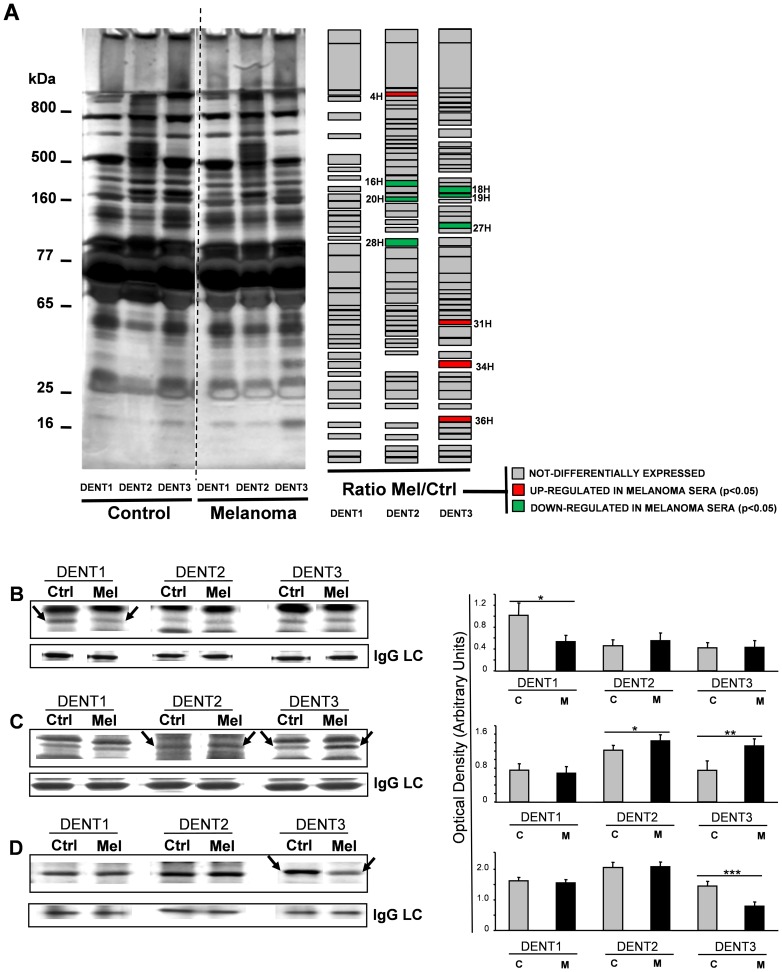
TRIDENT analysis of melanoma *vs* control sera. A: Silver staining of TRIDENT analysis of sera from melanoma patients and healthy subjects. The most significantly (p<0.05) modified bands, as assessed by densitometric quantification of 3 different experiments each of them carried out on human sera from 4 healthy and 4 melanoma affected individuals, are schematically highlighted as green (down-regulated in melanoma sera) or red (up-regulated in melanoma sera) boxes. Only 1 control subject and 1 melanoma patients are shown. B, C, D panels: Silver staining of three different bands whose expression resulted modulated in control (Ctrl) *vs* melanoma (Mel) mice sera. Statistical analysis was performed on groups of 6 mice per treatment. The reported bands, marked by arrows, are those found significantly differently expressed in cancer sera compared to controls, in at least 3 independent experiments. B: Band M21 (Complement factor B) is down-regulated in sera from melanoma affected mice, in DENT1 condition, compared to the control (* = p<0.0476). C: Band M32 (Apo E) is up- regulated in sera from melanoma affected mice compared to the control, both in DENT2 (* = p<0.04083) and in DENT3 condition (** = p<0.0056). D: Band M15 (α2Macroglobulin) is down- regulated in sera from melanoma affected mice in DENT3 condition, compared to the control (*** = p<0.0074).

To investigate whether TRIDENT analysis of serum proteins may help to highlight any difference between sera from healthy and from human patients, this protocol was applied individually to sera from 10 melanoma patients compared to 10 control healthy subjects (Fig. 4A). Protein bands were quantified and those differently expressed in 3 independent experiments (p<0.05) were cut, digested and subjected to MALDI-TOF analysis for protein identification. In Table 4, 10 differentially detected protein bands, 4 up-regulated and 6 down-regulated in melanoma sera when compared to control sera, are reported. The unequivocal identification of these proteins was further confirmed by LC-MS/MS, as reported in Table 5.

#### Mouse melanoma model study

The TRIDENT protocol was then applied to the study of murine sera from 4 independent groups of 6 mice bearing cutaneous melanoma *vs* 4 independent 6 healthy mice, for a total of 48 mice. After TRIDENT procedure, 9 protein bands showed a significantly different expression between melanoma and healthy mice. Protein bands up- or down-expressed (p<0.05) in melanoma *vs* control mice sera were cut, digested and identified by MALDI-TOF/MS (Table 6). The electrophoretic separation of some bands, namely Band M21 (Complement factor B), Band M32 (Apo E) and Band M15 (α2MG) were shown in Figures 4B, C and D, and their densitometric quantification was reported (right panels). According to data reported in Table 4, Table 5 and Table 6, and Fig. 4B, C and D, α2MG was reproducibly down-regulated in both human and mice melanoma *vs* healthy controls, while two lipoproteins (Apo E and Apo A1) were reproducibly up-regulated, in both human and mice melanoma *vs* healthy controls.

### Immunological validation

**Figure 5:**
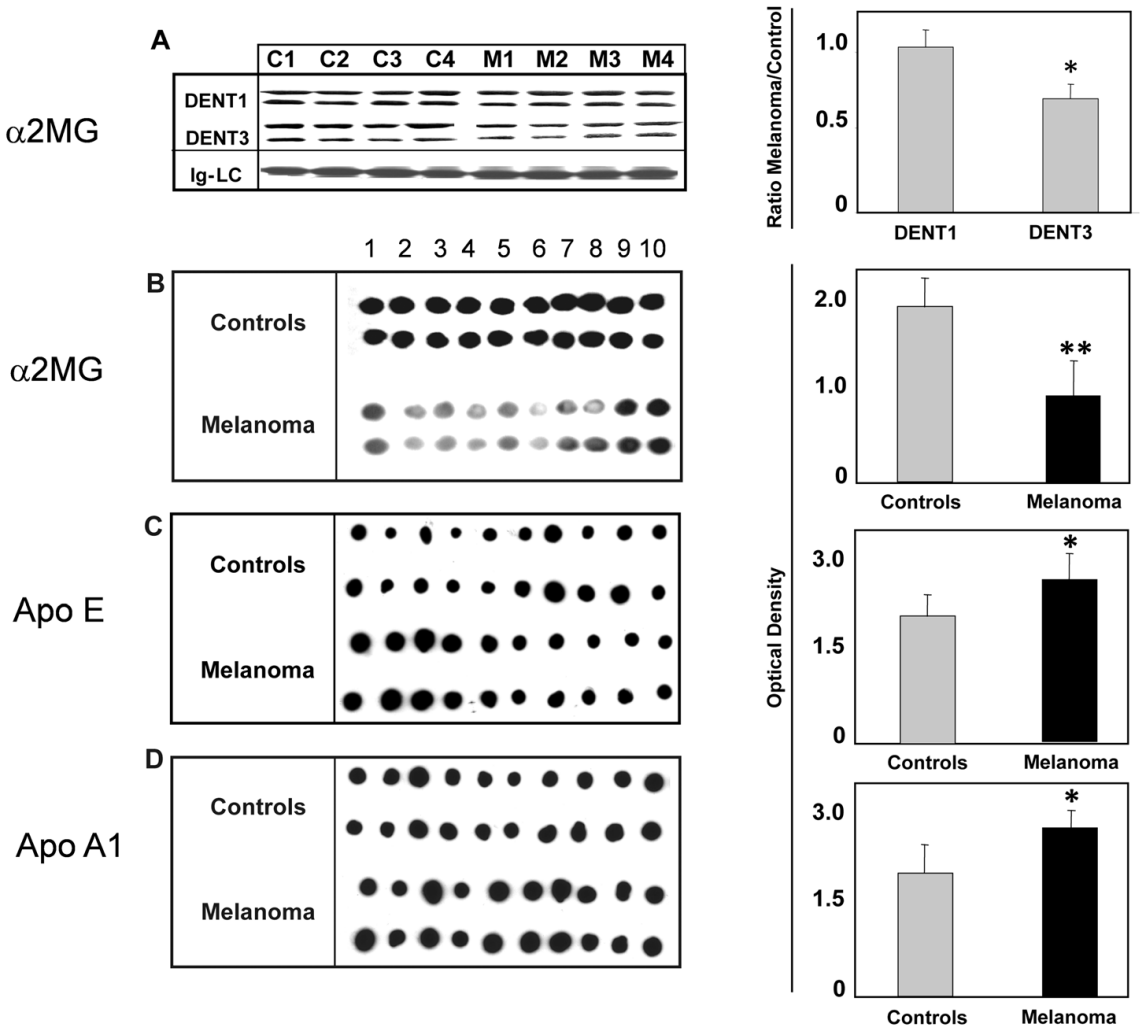
Immunological validation of α2MB, Apo E and Apo A1 as potential diagnostic biomarkers. A: Representative WB with anti-α2MB on human sera from 4 healthy (C) and 4 melanoma (M) affected individuals. The sera were pre-treated with the TRIDENT protocol, fractionated on gradient manually poured gels and submitted to electro-blotting (see Methods). In the Figure only the DENT1 and DENT3 protocols are shown. Each lane was loaded with 120 µg of serum proteins. Densitometric analysis (right panel), reports the ratio Melanoma/Control expression for the α2MG band, revealed as a doublet under this pre-treatment protocol (* = p<0.032). The same amount of protein was loaded per lane, as confirmed by comparing immunoglobulin light chain (Ig-LC) stained by Coomassie blue. B: Dot blot with anti-human α2MG on human sera from 10 healthy (Controls) and 10 melanoma affected individuals. The sera diluted 1∶5 with PBS were loaded in duplicate on nitrocellulose membrane (50 µg of total proteins per spot, equal amount of loaded proteins checked by Bradford assay and Ponceau Red) and submitted to primary anti-human α2MG, then HRP-conjugated secondary antibodies incubation followed by ECL detection (** = p<0.001). Densitometric analysis (right panel) reports the evaluation of α2MB expression in controls and melanoma sera. Data are reported as means ± SD. C and D: Dot blot with anti-human Apo E and Apo A1, respectively, carried out as above described (* = p<0.04).

To verify the results of TRIDENT protocol, we analyzed human α2MG expression in melanoma sera by two independent immunological methods, Western Blot (WB) and Dot Blot (DB) analyses, using two different commercial antibodies raised against human α2MG. Both Figure 5A (WB) and Figure 5B (DB) confirmed the down-modulation of serum α2MG in melanoma patients observed by SDS-PAGE. Further, the different expression of Apo E and Apo A1 in melanoma *vs* control sera was also confirmed by DB analyses, showing that both proteins were up-regulated in melanoma sera when compared to controls (Figures 5C and 5D, respectively).

## Discussion

Serum is the most abundant source of information for human disease diagnosis, including cancer; however, despite many efforts carried out, only a few molecular signals identified in sera are currently useful for diagnostic or prognostic purposes. The field of research focused on serum biomarker discovery was recently subjected to a critical review [2], [23]. In fact, while a very large number of potential serum biomarkers were suggested in hundreds of studies, only a few of them overcame validation and reproducibility issues to achieve clinical application. Several reasons underlie such failure, mainly linked to the complexity of the serum proteome, difficulties to achieve optimal serum protein solubilisation, presence of carrier proteins (e.g. albumin and immunoglobulins, in large excess over any other protein, which often carry other smaller signals) which represent a further complication for proteomic analyses. Depletion of such proteins may be necessary but, due to the complexity of the serum itself, e.g. the presence of lipids, often the depletion step may represents a source of additional reproducibility issues. In fact, removing cargo proteins may lead the undesired removal of the carried signals too. Therefore in the present study we set up a procedure to analyze the whole serum proteome without any depletion step. This, while makes the study more complex, on the other hand allows to give information not only regarding the presence of proteins in human serum, but also regarding their ability to interact with others, an important issue related to protein solubility and folding. To minimize the individual serum proteomic differences, pool of several sera from healthy subjects were analyzed in preliminary experiments, then to confirm the obtained results and to investigate any potentially relevant difference, several sera from individual cancer patients and control subjects were evaluated simultaneously. The novel procedure described in the present study, called TRIDENT, consists of the application of three different denaturation protocols to the same sera samples. The samples are electrophoretically separated simultaneously by SDS-PAGE and each protein pattern compared to the others, leading to a significant improvement of band resolution as compared to the standard procedure and giving a more complex and detailed protein pattern. To optimize this procedure, we tested many denaturation protocols and other factors such as polyacrylamide gradient and time length of electrophoretic separation. Optimal results were achieved using 16×18 cm slab gels with a continuous gradient of 2.4–15% acrylamide-bisacrylamide solution, followed by silver staining detection. It is noteworthy that, due to the different chemical-physical pre-treatments used, each protein band profile should be considered distinct from another derived from the same sample undergoing a completely different denaturation protocol; in fact, different denaturation protocols may differently affect protein unfolding and therefore protein electrophoretic resolution. The 2 denaturation protocols showing the highest protein bands discrimination (DENT2 and DENT3) were chosen among 69 different protocols and compared to the standard Laemmli denaturation protocol (DENT1). Such differential denaturation protocol was called TRIDENT and then applied to a number of melanoma patients sera and compared with control sera and let to the identification of proteins differentially detectable in cancer *vs* healthy sera. Such differential detection of serum proteins may be due to different expression level and to different “*denaturability*”, i.e. sensitivity to unfolding agents, of patient sera proteins when compared to healthy sera proteins. Such differences may be due, for instance, to different post-translational modifications, hydrophobic interactions, hydrogen bonds and other weak interactions with other proteins, including abundant carrier proteins, potentially related to specific pathological status. It is noteworthy that among the serum proteins separated by TRIDENT-SDS-PAGE, we were able to identify some proteins (at least one protein, namely IGHG1) usually not detected by conventional SDS-PAGE of serum without any depletion or enrichment pre-treatment. This is likely due to the ability of the described differential denaturation protocol to unfold proteins otherwise “buried” in large complexes blocked into the stacking gel or not able to enter the gel itself. Further, the comparison among different denaturation protocols may unmask and highlight faint differences. This suggests that serum and plasma contain cargos, membranes and multimeric complexes still underestimated and poorly studied, whose investigation deserves novel methodological approaches. The improvement due to the serum protein differential denaturation was also confirmed by LC-MS/MS analysis of a fraction of electrophoretically separated serum proteins, i.e. a small gel fragment migrating at electrophoretic position higher than 98 kDa. Therefore we believe the additional denaturation achieved via TRIDENT protocol may improve the analysis of serum proteins or other complex protein mixtures. Further, TRIDENT protocol allows the simultaneous run of many samples onto the same gel, making easier and quicker the comparison of different samples. On the other hand, 2D gel analysis may also be implemented within the TRIDENT protocol, to achieve even better results. As shown in Figures 2 and 3, the same protein band from identical serum samples may be differently stained according to DENT1, DENT2 or DENT3 denaturation protocol. This was observed in a reproducible manner in silver stained gels, suggesting that each protein band could be quantitatively and/or qualitatively different depending on the DENT used. The total number of detectable protein bands could be further increased if the differential denaturation protocol is carried out with more than 3 DENTs, see for instance Figure 2A where the pooled serum was denatured under 5 different protocols and a total of more than 200 different bands was discriminated. The first interesting result of the present study was therefore the finding that serum proteome contains a significant number of proteins whose detection is closely dependent on protein sensitivity to denaturing agents, currently underestimated with a single denaturation step. The detection of these “hidden” proteins is improved by the comparison among different denaturation treatments which the same serum was exposed to. TRIDENT protocol was then applied to a specific cancer type, i.e. sera from mice bearing cutaneous melanoma at early stage compared to sera from control mice and sera from early-non-metastatic skin melanoma human patients compared to sera from healthy individuals. Cutaneous melanoma is characterized by high aggressiveness, early metastatic dissemination and poor prognosis in the metastatic stage. Several circulating biomarkers in melanoma patients have been identified by different approaches, including proteomic analyses. For instance, S100B, C reactive protein (CRP), lactate dehydrogenase (LDH), pro-platelet basic protein precursor PPBP and IL-8 may help to determine the prognosis of melanoma patients [24], [25]. However, most of these and other serum molecules fail to predict tumor progression or tumor recurrence. Therefore, melanoma patients urgently need valid serological tools to reach diagnosis and predict prognosis, since presently the only markers with a prognostic value in stage I–III are the histomorphological features of the primary tumor. Recently, serum amyloid A was proposed as a prognostic marker in melanoma by MS-based proteomic profiling using hydrophobic C18 surfaced magnetic beads [26] and Bak was proposed as a candidate melanoma biomarker by using Hydrogel nanoparticles to analyze the low molecular weight serum proteins [27]; likely other recently developed techniques with promising potential may further improve the *scenario* [28]. When TRIDENT protocol was applied to melanoma and control sera, 3 proteins were found to be reproducibly and significantly up- or down-regulated in cancer serum proteome compared to controls in both murine and human sera (see Tables 4, 5 and 6). Further, it has been recently demonstrated that oncogenic pathways may stimulate production of vesicular structures (microparticles) released into the blood-stream from cells upon activation, malignant transformation, stress or death [29]. One of the most significantly modified proteins, down-regulated in melanoma, was identified as α2MG, a potent protease inhibitor able to modulate several cellular processes, including cell adhesion, proliferation, migration and invasion, key processes involved in cancer progression [30]–. Other significantly up-regulated proteins in cancer *vs* control sera, in both murine and human cancer, were Apo E and Apo 1. Therefore, as a consequence of the application of the new developed TRIDENT protocol, a novel diagnostic protein signature for human melanoma can be hypothesized. Other less abundant serum proteins were also found to be modulatedin murine or human melanoma alone (see Table 2 and Tables 4,5 and 6) confirming the TRIDENT efficacy may be further improved if combined with other techniques aimed at amplifying low-abundance proteins recovery. A validation study performed by immunological analyses on human sera from melanoma and controls subjects confirmed that the proteins differentially expressed in sera from cutaneous melanoma patients were human α2MG, Apo E and Apo 1 with statistically significant differences. TRIDENT protocol was developed to optimize the protein electrophoretic discrimination of whole serum proteome, but its ability to improve protein bands discrimination in complex mixtures of proteins like cell or tissue extracts was also confirmed by using B16-F10 melanoma cell protein extracts (data not shown). Therefore, the different sensitivity to denaturation of proteins or protein-complexes, may reveal structural modifications due for instance to post-translational processes or protein/protein interactions otherwise difficult to be evidenced by conventional techniques. Thus, a differential denaturation protocol such as TRIDENT may significantly increase the protein electrophoretic discrimination by highlighting small but reproducibly relevant differences in a complex proteome like the one carried by the serum. The down-regulation of α2MG detected in sera from melanoma patients in the present study is a novel finding although not surprising and in agreement with reported data showing that cancer cell adhesion and invasiveness is directly related to the activation of metalloproteinases [31] and the known potent activity of α2MG as metalloproteinases enzymatic activity inhibitor [32]. The involvement of α2MG in aggressiveness of human cancer cells was hypothesized by *in vitro* studies [32]–[35], but the results of the present study represent the first *in vivo* evidence from a murine melanoma model confirmed in human patients, suggesting α2MG, Apo E and Apo A1 as novel potential serum predictors in cutaneous melanoma patients. This may open new interesting perspectives to investigate cancer pathogenetic pathways and to improve the study of complex protein mixtures.

In conclusion, the results reported in the present study, starting from a new methodological approach, represent: a) the first evidence that serum contains a large amount of protein information, currently underestimated according to a single denaturation step, indicating a novel strategy for further biomarkers discovery based on the comparison of differentially denatured samples; b) the first *in vivo* evidence about a possible role of α2MG, Apo E and Apo A1 as early diagnostic predictors in cutaneous melanoma patients to be further investigated.

## Supporting Information

Table S1 **Pre-treatments tested on serum samples.** Description of the pre-treatments (PT, physical, chemical and combinations of them) tested on human and commercial bovine sera (from Sigma Aldrich). The name of the 3 PTs selected for the TRIDENT analysis are in bold.
(DOC)

Table S2 **Proteins identified by LC-MS/MS of a >98 kDa electrophoretic band.** The green boxes indicate the proteins identified by our TRIDENT-LC-MS/MS analysis never before detected in normal serum/plasma samples.
(XLSX)
