# Supplementary material for: Differential Denaturation of Serum Proteome Reveals a Significant Amount of Hidden Information in Complex Mixtures of Proteins
Source: PLoS One. 2013 Mar 22;8(3):e57104. doi: 10.1371/journal.pone.0057104 (PMC3606341; doi:10.1371/journal.pone.0057104)
Supplement: Table S1 — Pre-treatments tested on serum samples. Description of the pre-treatments (PT, physical, chemical and combinations of them) tested on human and commercial bovine sera (from Sigma Aldrich). The name of the 3 PTs selected for the TRIDENT analysis are in bold. (DOC) [file pone.0057104.s001.doc]

**Supplementary** **Table S1**

Description of protocols

| **Name** | **Type of human / bovine serum pre-treatments** | **Description of serum denaturing pre-treatment** |
| --- | --- | --- |
| **PT-1** | **Reference pre-treatment** | Dilution 1:1 with bi-distilled water. |
| PT-2  PT-3  PT-4  PT-5 | **high salt pre-treatments** | Dilution 1:1 with solution of Sodium Chloride to reach a final concentration between 0.5 and 5 M (0.5 M =PT-2, 1 M =PT-3, 2.5 M =PT-4 and 5 M =PT-5). |
| PT-6  PT-7  PT-8  PT-9 | **high salt pre-treatments** | Dilution 1:1 with solution of ammonium bicarbonate to reach a final concentration between 0.5 and 5 M (0.5 M =PT-6, 1 M =PT-7, 2.5 M =PT-8 and 5 M =PT-9). |
| PT-10  PT-11 | **physical pre-treatments** | Dilution 1:1 with PBS followed by heating at 37 °C for 1 (PT-10) or 3 days (PT-11) |
| PT-12  PT-13 | **physical pre-treatments** | Dilution 1:1 with PBS followed by 3 (PT-12) or 10 cycles of freeze and thaw (PT-13). |
| **PT-14**  PT-15  PT-16 | **physical pre-treatments** | Dilution 1:1 with PBS followed by boiling at 100 °C for a period of time varying between 2.5 and 10 min, centrifuge at 10,000 g for 15 min, room temperature, with subsequent careful pellet recovery (2.5 min = PT-14, 5 min = PT-15, 10 min = PT-16). |
| PT-17  PT-18  PT-19 | **physical pre-treatments** | Dilution 1:1 with PBS followed by boiling at 100 °C for different times between 2.5 and 10 min with subsequent supernatant recovery (2.5 min = PT-17, 5 min = PT-18, 10 min = PT-19). |
| PT-20  PT-21  PT-22 | **physical pre-treatments** | Dilution 1:1 with PBS followed by ultra-filtration with 3, 10, 30 kDa cut off membranes (3 kDa = PT-20, 10 kDa = PT-21, 30 kDa = PT-22). |
| PT-23  PT-24  PT-25 | **detergent pre-treatments** | Dilution 1:1 with detergent (Nonidet P40) to reach 1, 1.5 or 2% final concentration (1% = PT-23, 1.5% = PT-24, 2% = PT-25). |
| PT-26  PT-27  PT-28 | **detergent pre-treatments** | Dilution 1:1 with detergent (Triton X100) to reach 1, 1.5 or 2% final concentration (1% = PT-26, 1.5% = PT-27, 2% = PT-28). |
| PT-29  PT-30  PT-31 | **detergent pre-treatments** | Dilution 1:1 with detergent (Tween 20) to reach 1, 1.5 or 2% final concentration (1% = PT-29, 1.5% = PT-30, 2% = PT-31). |
| PT-32  PT-33  PT-34  PT-35 | **chemical pre-treatments** | Dilution 1:1 with HSSB with 2−β mercaptoethanol (2-βMSH) at 1, 2.5, 5, 10% (v/v) final concentration (1% = PT-32, 2.5%=PT-33, 5%=PT-34, 10%=T-35). |
| PT-36 | **chemical pre-treatment** | Dilution 1:1 with HSSB without 2-βMSH, followed by boiling at 100 °C for 2.5 min. |
| PT-37  PT-38  PT-39  PT-40 | **chemical/physical pre-treatments** | Dilution 1:1 with Sodium chloride at 1 M final concentration followed by boiling at 100 °C for different period of time (1 min =PT-37, 2.5 min = PT-38, 5 min = PT-39, 10 min = PT-40). |
| PT-41  PT-42  PT-43  PT-44 | **chemical/physical pre-treatments** | Dilution 1:1 with Sodium chloride at different concentration (0, 0.5, 1, 2 M) followed by ultra-filtration (no NaCl = PT-41, 0.5M = PT-42, 1M = PT-43, 2M =PT-44). |
| PT-45  PT-46 | **high temperature pre-treatments** | Dilution 1:1 with HSSB (without 2-βMSH) followed by boiling at 100 °C for 2.5 = PT- 45 or 5 min = PT-46 (pellet recovery). |
| PT-47  PT-48  PT-49 | **high temperature pre-treatments** | Dilution 1:1 with HSSB (5% 2-βMSH), followed by boiling at 100 °C for 2.5 = PT-47, 5 = PT-48 or 10 min = PT 49 (pellet recovery). |
| PT-50  PT-51  PT-52 | **strong reducing / detergent and high temperature pre-treatments** | Dilution 1:1 with HSSB (10% 2-βMSH), followed by boiling at 100 °C for 2.5 = PT-50, 5 = PT-51 or 10 min = PT 52 (pellet recovery). |
| PT-53  PT-54  PT-55 | **chemical and physical pre-treatments** | Dilution 1:1 with HSSB (5% 2-βMSH for 5, 10 or 20 min at 37 °C) followed by 0.45 μm filtration to remove large aggregates: 5 min = PT-53, 10 min = PT-54, 20 min = PT-55. |
| PT-56  PT-57  PT-58 | **chemical and physical pre-treatments** | Dilution 1:1 with HSSB (10% 2-βMSH for 5, 10 or 20 min at 37 °C) followed by 0.45 μm filtration: 5 min = PT-56, 10 min = PT-57, 20 min = PT-58. |
| PT-59 | **chemical and physical pre-treatment** | Dilution 1:1 with HSSB (without 2-βMSH) followed by filtration. |
| PT-60  PT-61 | **short chemical and physical pre-treatments** | dilution 1:1 with sonication buffer (SB), followed by sonication for 10 min then followed by dilution 1:1 with HSSB (5% 2-βMSH) and boiling at 100 °C for 2.5 = PT-60 or 5 min = PT-61. |
| PT-62  PT-63 | **longer chemical and stronger physical pre-treatments** | dilution 1:1 with SB, followed by sonication for 30 min then followed by dilution 1:1 with HSSB (5% 2-βMSH) and boiling at 100 °C for 2.5 =PT-62 or 5 min = PT-63. |
| **PT-64**  PT-65 | **longer chemical and stronger physical pre-treatments** | dilution 1:1 with SB, followed by sonication for 60 min then followed by dilution 1:1 with HSSB (5% 2-βMSH) and boiling at 100 °C for 2.5 =PT-64 or 5 min = PT-65. |
| PT-66  PT-67 | **longer chemical and stronger physical pre-treatments** | Serum sonication for 90 min at room temperature; then dilution 1:1 with HSSB (5% 2-βMSH) followed by boiling at 100 °C for 5 = PT-66 or 10 min = PT-67 |
| PT-68  PT-69 | **longer chemical and stronger physical pre-treatments** | Serum sonication for 90 min at 38 °C; then dilution 1:1 with HSSB (5% 2-βMSH) followed by boiling at 100 °C for 5 = PT-68 or 10 min = PT-69 |
